# The association between variants in *PLA2R* and *HLA-DQA1* and renal outcomes in patients with primary membranous nephropathy in Western China

**DOI:** 10.1186/s12920-021-00969-0

**Published:** 2021-05-08

**Authors:** Shulei Fan, Qiuxia Wang, Amanda Y. Wang, Ping Zhang, Xiang Zhong, Shasha Chen, Guisen Li, Li Wang, Wei Wang

**Affiliations:** 1 Department of Nephrology and Institute of Nephrology, Sichuan Academy of Medical Science and Sichuan Provincial People’s Hospital, Chengdu, 610072 China; 2 The Renal and Metabolic Division, The George Institute for Global Health, University of NSW, Sydney, Australia; 3 The Department of Renal Medicine, Concord Repatriation General Hospital, Concord, NSW Australia; 4 The Concord Clinical School, University of Sydney, Camperdown, Australia; 5 Department of Respiratory Medicine, The Second Affiliated Hospital of Chongqing Medical University, Chongqing, China

**Keywords:** Primary membranous nephropathy (PMN), M-type phospholipase A2 receptor (*PLA2R*), Human leukocyte antigen complex class II HLA-DQα-chain 1 (*HLA-DQA1*), Genotype, Renal outcome

## Abstract

**Background:**

Both Genome-wide associations and our previous study have shown that single nucleotide polymorphisms (SNPs) of M-type phospholipase A2 receptor (*PLA2R*) and human leukocyte antigen complex class II HLA-DQα-chain 1 (*HLA-DQA1*) gene were identified to be associated with primary membranous nephropathy (PMN). However, whether these SNPs affect clinical manifestation and renal outcome for PMN patients is poorly defined. Here, we evaluated whether there is an association between these SNPs and clinical manifestations and renal outcomes of PMN in a western Chinese cohort.

**Methods:**

Seven SNPs within *PLA2R* and one SNP in *HLA-DQA1* were selected in our study. Clinical data from 314 patients with PMN were collected and the relationship between the genotype and phenotype was evaluated. A total of 186 patients had follow-up data. We assessed the treatment responses and renal outcomes between patients with these gene polymorphisms after a median follow-up of 18.6 months.

**Results:**

Eight SNPs were not associated with clinical manifestations of PMN patients (Pc < 0.05). rs3828323 T allele was marginally significantly associated with hypertension (*P* = 0.008, *Pc* = 0.064, OR = 1.821). After treatment for PMN, the SR group (including CR and PR) had lower serum creatinine level (68.4 ± 18.8 μmol/L vs. 122.8 ± 126.6 μmol/L, *P* < 0.001), urea (5.5 ± 1.9 mmol/L vs. 8.0 ± 4.0 mmol/L, *P* < 0.001), uric acid (358.5 ± 95.1 μmol/L vs. 392.8 ± 118.1 μmol/L, *P* = 0.037) and urinary protein (0.23 (0.76,1.05) g/d vs. 3.01 (2.06,7.95) g/d, *P* < 0.001), higher eGFR (100.0 ± 20.1 ml/min/1.73m^2^ vs. 77.1 ± 35.3 ml/min/1.73m^2^, *P* < 0.001) and albumin (41.1 ± 5.1 g/L vs.30.4 ± 8.2 g/L, *P* < 0.001). We also identified that PMN patients with CT/TT genotype for rs3828323 achieved higher cumulative survival rate than patients with CC genotype.

**Conclusions:**

Rs3828323 may influence hypertension and renal outcome in patients with PMN. Further research is needed to explore the mechanism for this genotype-disease phenotype association.

**Supplementary Information:**

The online version contains supplementary material available at 10.1186/s12920-021-00969-0.

## Background

Primary membranous nephropathy (PMN) is one of the most common causes for nephrotic syndrome for adult. Its typical pathological features are diffuse thickening of glomerular capillary basement membrane and deposition of subepithelial immune complexes [1, 2]. In recent years, its incidence is increasing. It is the second most common type of primary glomerulonephritis in China [3, 4].

PMN is a typical kidney disease caused by antigen–antibody reaction. Antigen–antibody binding forms immune complex, then activates complement system, resulting in injury of podocytes and glomerular basement membrane, and eventually leading to kidney injury [5, 6]. The production of circulating *PLA2R* antibodies may be the main pathogenic mechanism of the disease [7]. The anti-*PLA2R* antibody has high specificity and good sensitivity. *PLA2R* antibody are positive in 70–80% of patients with PMN [8]. The higher the antibody titer is, the higher the risk of deterioration of renal function is [9, 10]. It is reported that some clinical factors are related to the progress of the disease, including severe proteinuria, hypertension, and renal dysfunction at diagnosis [11, 12].

In Caucasian population, genome-wide association studies have confirmed the susceptibility of *PLA2R* and *HLA-DQA1* gene with PMN [13]. Multiple loci in *PLA2R* and *HLA-DQA1* were closely related to PMN in various ethnicities, but the results of different regions and ethnic groups were not entirely consistent [8, 13–18]. To verify the previous findings in Western China, in our previous study we selected eight SNPs reported in the literatures and found that two SNPs (rs2715918, rs4665143) within *PLA2R*, 1 SNP in *HLA-DQA1* (rs2187668) were associated with primary membranous nephropathy [19]. The interactions of rs2715918, rs4665143 and rs2187668 were associated with an increased risk of the development of PMN by10.61-fold. Patients carrying risk alleles confer a predisposition to anti-*PLA2R* autoantibody generation [19]. In a Spanish study, Bullich et al. found that PMN patients with two SNP risk alleles, rs2187668 and rs4664308, had better response to immunosuppressive therapy and slower progression of chronic kidney disease [17]. Wang et al. found that PMN Patients with HLA-DRB1*1502 had worse kidney outcomes in Han Chinese [20].

To evaluate whether these SNPs are associated with clinical manifestations and renal outcomes of PMN patients, clinical data from 314 patients with PMN were collected and the relationship between the genotype and phenotype was evaluated. We performed a retrospective cohort study of 186 patients who had follow-up data to assess the relationship between genetic polymorphisms and renal outcome.

## Methods

### Patients

From January 2010 to December 2016, patients with biopsy-proven PMN in Sichuan Provincial People’s Hospital were recruited. Among them, 314 patients who had complete clinical baseline data in our Renal Treatment System (RTS) database were included in the study. All the patients came from Western Han ethnicity. In addition, the data on complete follow-up more than 3 months were available and recorded in 186 patients. Patients with membranous nephropathy due to secondary causes, such as systemic lupus erythematosus, cancer, hepatitis B virus infection and drug were excluded.

The study was approved by the Ethics Committee of the Sichuan Provincial People’s Hospital (Chengdu, China), and all the patients signed informed consents to participate in this study.

### Genotyping of *PLA2R *and *HLA-DQA1*

The methods for selection of candidate SNPs of *PLA2R* and *HLA-DQA1* and genotyping were described in the previous study [19]. SNPs of the candidate genes were obtained from previously published polymorphisms associated with PMN.

### Genotype–phenotype correlation studies

Baseline clinical data were collected from all patients at diagnosis. PMN patients were divided into several subgroups based on the following parameters: 24 h urinary protein excretion (24 h-u-pro ≥ 3.5 g/d or < 3.5 g/d), renal function (eGFR ≤ 60 or > 60 mL/min/1.73 m^2^), and blood pressure (≥ 140/90 mmHg or < 140/90 mmHg). The eGFR calculation was using the CKD-EPI equation [21]. The association of alleles frequencies, genotype frequencies and different genetic models with clinical phenotype (24-h-pro, eGFR and blood pressure) in PMN patients was analyzed.

### Detection *PLA2R* antibody in serum

One hundred and twenty PMN patients had anti-*PLA2R* antibodies detected to analyze the relationship between anti-*PLA2R* positivity and treatment response, as well as renal outcomes. The methods for measuring *PLA2R* antibody in serum were described in the previous study [19].

### Definition

ESRD was defined as eGFR < 15 mL/min/1.73 m^2^, receiving dialysis therapy for more than 3 months or transplantation [22]. The primary outcome was renal progression, defined as a composite of ESRD, a reduction in eGFR by > 30% from the baseline and doubling serum creatinine. Spontaneous remission (SR) included complete remission and partial remission. Complete remission (CR) was defined as 24 h urinary protein excretion < 500 mg per day with normal serum creatinine and serum albumin (≥ 35 g/L). Partial remission (PR) was defined as 24 h urinary protein excretion of 0.5–2 g per day or < 50% of baseline with normal serum creatinine and serum albumin level [23]. Patients who did not have complete or partial remission were called non- spontaneous remission. According our previous study, the *PLA2R* low-risk was defined as individuals with GG in rs2715918 and GG in rs4665143 within *PLA2R*. The rest were the *PLA2R* high-risk group. The *HLA-DQA1* low-risk was defined as individuals with GG in rs2187668 within *HLA-DQA1*. The rest were the *HLA-DQA1* high-risk group [19].

### Statistical analysis

Continuous variables were presented as mean and standard deviation quartile spacing standard deviation (SD). Categorical variables were presented as median with quartiles. Chi-square test was used to compare the classified variables between the two groups. Categorical variables were presented by proportions. Comparisons of continuous variables using t-test or one-way ANOVA. Hardy–Weinberg equilibrium (HWE) was determined by chi-square test. Logistic regression analysis was used to test three kinds of models of inheritance. Bonferroni adjustment was performed to correct P values (Pc). The association between gene mutation and kidney survival was analyzed by Kaplan–Meier.

## Results

### Baseline characteristics of PMN patients

The baseline characteristics of 314 (166 male and 148 female) PMN patients were described in Table 1. All those who participated in the study lived in Sichuan province for several generations and belonged to the Han ethnicity. The mean age at the time of renal biopsy was 49.1 ± 13.5 years old, and the mean eGFR was 101.33 ± 22.67 ml/min per 1.73 m^2^. The median baseline proteinuria was 4.03 (quartiles,2.34–6.20) g/24 h.

**Table 1:** Baseline clinical characteristics of the study cohort

Characteristic	Patients with PMN (n = 314)
Male/female	166/148
Age at biopsy (year)	49.1 ± 13.5
Male (n, %)	166 (52.9)
Scr (μmol/L)	68.60 ± 26.80
Urea (mmol/L)	5.80 ± 4.11
eGFR (ml/ (min·1.73m^2^))	101.33 ± 22.67
Urine protein (g/24 h)	4.03 (2.34,6.20)
HGB (g/L)	131.18 ± 19.78
Alb (g/L)	27.20 ± 15.84
SBP (mmHg)	128.06 ± 15.83
DBP (mmHg)	78.23 ± 11.24
Hypertension (n, %)	78 (24.8)

### Genotype–phenotype correlations

Eight SNPs were successfully genotyped. The details of SNPs were shown in our pervious study [19]. All of them conformed to the Hardy–Weinberg equilibrium (HWE). We determined the association of alleles, genotype frequencies and different genetic models of candidate eights SNPs with phenotype (24 h-u-pro, eGFR, blood pressure, respectively) in 314 PMN patients (Additional file 1: Supplementary Tables S1–S3, respectively). The result revealed significant differences in alleles, genotype distributions, dominant, recessive, additive model of rs3828323 with hypertension (*P* = 0.008, 0.029, 0.037, 0.034, 0.012, respectively). After Bonferroni adjustment, there was no statistical significance in the above variables (*P* = 0.064, 0.232, 0.296, 0.272, 0.096, respectively). There was no association of alleles frequencies, genotype frequencies and different genetic models of rs3828323 with 24-h-pro or eGFR. No association was found between other SNPs and clinical phenotypes (24 h-u-pro, eGFR, blood pressure). These results indicated the potential effects of rs3828323 on hypertension.

### Follow-up data and treatment response

Among all the 314 individuals, 186 patients had complete follow-up data. Their baseline and follow-up characteristics were described in Table 2. Forty-one (22.0%) patients reached the composite outcome during a median follow-up time of 18.6 (quartiles, 6.7–45.5) months. The numbers of corticosteroid monotherapy, immunosuppression (Steroid + cyclophosphamide, Calcineurin inhibitor with or without steroid) and non-immunosuppression (with angiotensin-converting enzyme inhibitors or angiotensin II receptor blockers) were 29 (15.6%), 112 (60.2%), 45 (24.2%), respectively. The result also showed that among 112 patients receiving immunosuppressive therapy, the numbers of complete remission, partial remission and non-spontaneous remission were 37 (33.0%), 21 (18.8%), 54 (48.2%), respectively. Among 45 patients who did not receive immunosuppressive therapy, the numbers of complete remission, partial remission and non-spontaneous remission were 14 (31.1%), 13 (28.9%), 18 (40.0%), respectively (Table 2).

**Table 2:** Baseline and follow-up clinical characteristics of the study cohort

Characteristic	Patients with PMN (n = 186)
Baseline	
Age at biopsy (years)	49.1 ± 13.4
Male (n, %)	102 (54.8)
SCr (μmol/L)	68.9 ± 22.5
eGFR (ml/min/1.73 m^2^)	100.8 ± 20.7
Urea (mmol/L)	5.6 ± 2.2
Alb (g/L)	28.4 ± 19.1
UA (μmol/L)	362.0 ± 95.5
24 h-u-pro (g/d)	4.17 (2.31,7.34)
SBP (mmHg)	128.4 ± 15.2
DBP (mmHg)	78.5 ± 11.0
Hypertension (n, %)	47 (25.3)
Follow-up	
SCr (μmol/L)	92.4 ± 89.1
eGFR (ml/min/1.73 m^2^)	90.6 ± 29.9
Urea (mmol/L)	6.6 ± 3.2
Alb (g/L)	36.4 ± 8.5
UA (μmol/L)	373.3.0 ± 106.6
24 h-u-pro (g/d)	0.86 (0.15,2.65)
Follow-up time (month)	18.6 (6.7,45.5)
Endpoint event (n,%)	41 (22.0)
Therapy	
Corticosteroid monotherapy (n,%)	29 (15.6)
Immunosuppression (n,%)	112 (60.2)
Non- Immunosuppression (n,%)	45 (24.2)
Treatment effect	
Corticosteroid monotherapy	
CR/PR/non-SR (n,%)	12/6/11 (41.4, 20.7, 37.9)
Immunosuppression	
CR/PR/non-SR (n,%)	37/21/54 (33.0, 18.8, 48.2)
Non- Immunosuppression	
CR/PR/non-SR (n,%)	14/13/18 (31.1, 28.9, 40.0)

The Endpoint event was defined as a combination of ESRD, a 30% reduction of eGFR from baseline and double times increasing of creatinine

### Baseline and follow-up clinical characteristics in the remission group and non-remission group

Compared these follow-up data with the non-SR group, the SR group (including CR and PR) had lower serum creatinine level (68.4 ± 18.8 μmol/L vs. 122.8 ± 126.6 μmol/L, *P* < 0.001), urea (5.5 ± 1.9 mmol/L vs. 8.0 ± 4.0 mmol/L, *P* < 0.001)), uric acid (358.5 ± 95.1 μmol/L vs. 392.8 ± 118.1 μmol/L, *P* = 0.037) and urinary protein (0.23 (0.76,1.05) g/d vs. 3.01 (2.06,7.95) g/d, *P* < 0.001)), higher eGFR (100.0 ± 20.1 ml/min/1.73m^2^ vs. 77.1 ± 35.3 ml/min/1.73m^2^, *P* < 0.001)) and albumin (41.1 ± 5.1 g/L vs.30.4 ± 8.2 g/L, *P* < 0.001)). In addition, we also found that compared the baseline data with the non-SR group, the SR group had lower creatinine (64.8 ± 22.1 μmol/L vs. 74.0 ± 22.1 μmol/L, *P* = 0.006) and urea (5.2 ± 2.0 mmol/L vs.* 6.0* ± 2.3 mmol/L, *P* = 0.017); higher serum albumin level (31.0 ± 24.7 g/L vs. 25.1 ± 6.2 g/L, *P* = 0.038) and eGFR (103.8 ± 20.5 ml/min/1.73m^2^ vs. 97.0 ± 20.5 ml/min/1.73m^2^, *P* = 0.025) (Table 3).

**Table 3:** Baseline and follow-up clinical characteristics in the remission group and non-remission group

Characteristic	Remission (n = 104)	Non-Remission (n = 82)	*P*
Baseline			
Age at biopsy (years)	47.7 ± 13.0	50.8 ± 13.8	0.120
Male (n, %)	44 (42.3)	60 (73.1)	< 0.001
SCr (μmol/L)	64.8 ± 22.1	74.0 ± 22.1	0.006
eGFR (ml/min/1.73m^2^)	103.8 ± 20.5	97.0 ± 20.5	0.025
Urea (mmol/L)	5.2 ± 2.0	6.0 ± 2.3	0.017
Alb (g/L)	31.0 ± 24.7	25.1 ± 6.2	0.038
UA (μmol/L)	365.1 ± 104.9	368.0 ± 92.7	0.871
24 h-u-pro (g/d)	4.05 (1.97,6.31)	4.28 (2.49,8.66)	0.059
Hypertension (n, %)	30 (28.8)	22 (26.8)	0.761
Follow-up			
SCr (μmol/L)	68.4 ± 18.8	122.8 ± 126.6	< 0.001
eGFR (ml/min/1.73 m^2^)	100.0 ± 20.1	77.1 ± 35.3	< 0.001
Urea (mmol/L)	5.5 ± 1.9	8.0 ± 4.0	< 0.001
Alb (g/L)	41.1 ± 5.17	30.4 ± 8.24	< 0.001
UA (μmol/L)	358.5 ± 95.1	392.8 ± 118.1	0.037
24 h-u-pro (g/d)	0.23 (0.76,1.05)	3.01 (2.06,7.95)	< 0.001

### Genetic Variants and Immunosuppressive Therapy Response

Our previous studies showed interaction of rs2715918 GA/AA, rs4665143 GA/AA and rs2187668 GA/AA could significantly increase the risk for the development of PMN by 10.61-fold [19]. Therefore, in this study, we still choose these 3 SNPs and detected the association between genetic variants and response to immunosuppressive therapy. Genotypes under a dominant mode were considered the non-risk genotypes for PMN susceptibility and used as a reference. In univariate and multivariate logistic regression analysis, we found no statistical significance in response to immunosuppressive therapy between the reference and the genotypes carried risk alleles (Table 4).

**Table 4:** Logistic regression analyses between SNPs within *HLA-DQA1* and *PLA2R* genes and primary membranous nephropathy response to immunosuppressive therapy

RS#	Remission	Non- Remission	Univariate analysis^a^		Multivariate analysis^a,b^	
	n (%)	n (%)	OR (95%CI)	*P*	OR (95%CI)	*P*
rs2715918						
GG	39 (67.3)	38 (70.4)	1			
GA	17 (29.3)	13 (24.0)	0.864 (0.388–1.926)	0.721	1.273 (0.545–2.978)	0.577
AA	2 (3.4)	3 (5.6)				
rs4665143						
GG	15 (25.9)	9 (16.7)	1			
GA	20 (34.5	26 (48.1)	1.744 (0.691–4.403)	0.239	2.434 (0.870–6.809)	0.090
AA	23 (39.6)	19 (35.2)				
rs2187668						
GG	34 (58.6)	31 (57.4)	1			
GA	23 (39.7)	22 (40.7)	1.051 (0.496–2.227)	0.897	1.139 (0.521–2.494)	0.744
AA	1 (1.7)	1 (1.9)				

^a^Univariate analysis considering a dominant model for *HLA-DQA1* and a dominant model for *PLA2R*. The non-risk genotypes for primary membranous nephropathy susceptibility were considered as the reference. ^b^Adjusted for proteinuria at diagnosis

### Genetic variants and decline in renal function

**Fig. 1:**
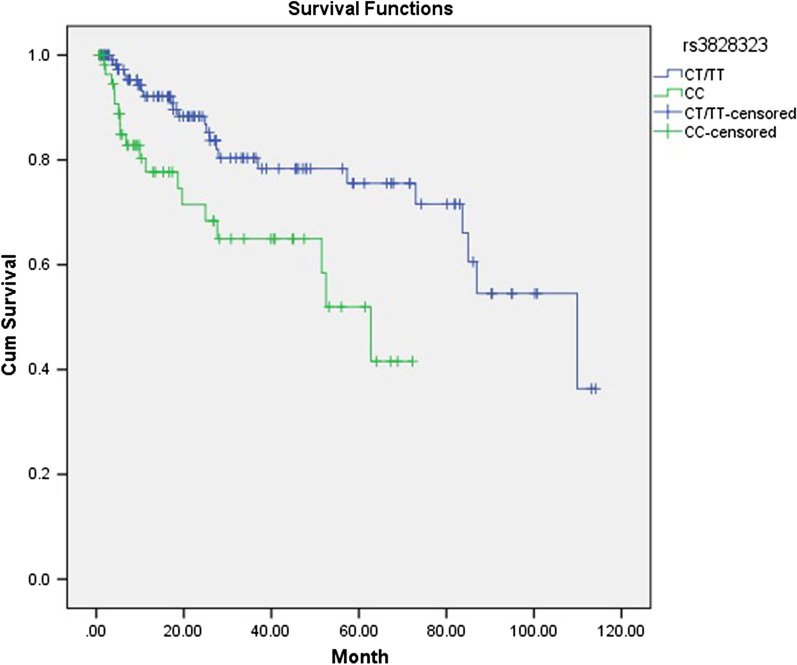
Survival analysis of time with outcome. Kaplan–Meier analysis showed that with the prolongation of follow-up time. **a** the CT/TT genotype for rs3828323 achieved higher cumulative survival rate than CC genotype (*P* = 0.003). **b** Subgroup analysis found that in the group of immunosuppression, the CT/TT genotype for rs3828323 achieved higher cumulative survival rate than CC genotype (*P* = 032). **c** For the rest of the patients, the difference was not statistically significant (*P* = 0.072)

Forty-one (22.0%) patients reached the composite outcome during a median follow-up time of 18.6 (quartiles, 6.7–45.5) months. Kaplan–Meier analysis showed that for rs3828323, patients carrying CT/TT genotype achieved superior outcomes than CC genotype (*P* = 0.003, Fig. 1a). We also performed subgroup analysis and found that in the group of immunosuppression (n = 112), the CT/TT genotype for rs3828323 achieved higher cumulative survival rate than CC genotype (*P* = 032, Fig. 1b). For the rest of the patients (n = 76), the difference was not statistically significant (*P* = 0.072, Fig. 1c). No associations were found with other SNPs.

### Association between *PLA2R*/*HLA-DQA1* Genotype and Anti-*PLA2R* Antibody

Our study included 314 PMN patients, of which 120 patients had anti-*PLA2R* antibodies in plasma tested. According to our previous study, we found that patients with low-risk SNPs had lower anti-*PLA2R* antibody positive rate than patients with high-risk SNPs [19]. Furthermore, we found there was no statistically significant difference in baseline characteristics between anti-*PLA2R* antibody positive group and anti-*PLA2R* antibody negative group (Table 5). Table 6 listed baseline characteristics in different combination groups. Both *PLA2R* and *HLA-DQA1* high-risk group had the highest anti-*PLA2R* antibody positive rate (Table 6).

**Table 5:** clinical characteristics of PMN patients who detected circulating anti-*PLA2R* antibodies

Characteristic	anti-*PLA2R* ( +)	anti-*PLA2R* (-)	*P*
Baseline	n = 91	n = 29	
Age at biopsy (years)	51.07 ± 12.66	48.0 ± 15.63	0.172
Male (n, %)	57 (62.2)	15 (51.7)	0.296
Cr (μmol/L)	73.17 ± 25.05	75.60 ± 52.47	0.736
eGFR (ml/min/1.73 m^2^)	95.77 ± 21.78	100.84 ± 27.78	0.311
Urea (mmol/L)	6.17 ± 4.32	5.55 ± 2.71	0.472
Alb (g/L)	27.97 ± 26.29	27.7 ± 7.64	0.959
24 h-u-pro (g/d)	5.08 ± 3.55	4.36 ± 5.75	0.436
SBP (mmHg)	131.96 ± 15.16	127.89 ± 15.77	0.222
DBP (mmHg)	79.55 ± 10.68	78.39 ± 10.22	0.614

**Table 6:** Baseline characteristics of 120 PMN patients for detecting circulating anti-*PLA2R* antibody

	*PLA2R* low risk + HLA low risk	*PLA2R* low risk + HLA high risk	*PLA2R* high risk + HLA low risk	*PLA2R* high risk + HLA high risk	*P*
	n = 12	n = 7	n = 60	n = 41	
Male/female	8/4	4/3	36/24	24/17	0.963
Age (year)	48.33 ± 15.47	54.86 ± 15.08	49.95 ± 15.28	52.71 ± 9.30	0.564
Scr (μmol/L)	79.75 ± 28.70	73.78 ± 22.95	72.87 ± 39.41	73.30 ± 27.08	0.935
Urea (mmol/L)	5.78 ± 1.90	6.08 ± 2.74	6.43 ± 5.19	5.48 ± 2.21	0.707
eGFR (ml/ (min·1.73m2))	94.49 ± 27.64	91.90 ± 27.57	99.98 ± 23.42	94.26 ± 21.48	0.577
Urine protein (g/24 h)	8.30 ± 7.67	3.30 ± 2.07	4.73 ± 3.68	4.38 ± 3.09	0.010
HGB (g/L)	132.25 ± 17.11	132.14 ± 15.66	132.08 ± 15.72	133.07 ± 17.52	0.993
Alb (g/L)	23.11 ± 5.67	28.44 ± 7.23	25.74 ± 5.89	32.40 ± 38.65	0.465
anti-*PLA2R* ( +)/ ( −)	5/7	5/2	45/15	36/5	0.012
anti-*PLA2R* ( +) (%)	41.6	71.4	75.0	87.8	

## Discussion

In our retrospective study including 314 PMN patients, 8 SNPs of 2 genes (*PLA2R* and *HLA-DQA1*) were selected to analyze the association between SNPs and clinical phenotype in PMN patients. Rs3828323 T allele was marginally significantly associated with hypertension (*P* = 0.008, *Pc* = 0.064, OR = 1.821). Among 186 patients who had complete follow-up data, forty-one (22.0%) patients reached the composite outcome during a median follow-up time of 18.6 (quartiles, 6.7–45.5) months. The further study showed that patients carrying CT/TT genotype achieved better renal survival than patients carrying CC for rs3828323.The selected other candidate SNPs showed no association with clinical phenotype and renal outcomes in PMN patients.

Some of our results are consistent with the previous research. Liu et al. found that rs35771982 and rs6757188 had no significant correlation with different clinical manifestations of PMN in a retrospective study and they were not independent risk factors for disease progression [15]. Kim et al. identified that rs35771982 and rs3828323 were not associated with the prognosis of PMN patients [14]. However, in the current study, we found that patients with one or two T alleles (CT/TT) of rs3828323 had higher kidney survival than those without T allele (CC).

The natural course of PMN is diverse, with about one third of patients able to achieve spontaneous remission, about one third of patients with persistent proteinuria but long-term stable renal function, and another third of patients progressing slowly to end stage renal disease (ESRD) [11, 24, 25]. In our cohort, the median follow-up time of patients was 18.6 (quartiles, 6.7–45.5) months. 55.4% (n = 103) of them achieved spontaneous remission. Compared these follow-up data with the non-SR group, the SR group (including CR and PR) had lower serum creatinine level (68.4 ± 18.8 μmol/L vs. 122.8 ± 126.6 μmol/L, *P* < 0.001), urea (5.5 ± 1.9 mmol/L vs. 8.0 ± 4.0 mmol/L, *P* < 0.001)), uric acid (358.5 ± 95.1 μmol/L vs. 392.8 ± 118.1 μmol/L, *P* = 0.037) and urinary protein (0.23 (0.76,1.05) g/d vs. 3.01 (2.06,7.95) g/d, *P* < 0.001)), higher eGFR (100.0 ± 20.1 ml/min/1.73m^2^ vs. 77.1 ± 35.3 ml/min/1.73m^2^, *P* < 0.001)) and albumin (41.1 ± 5.1 g/L vs.30.4 ± 8.2 g/L, *P* < 0.001)). In addition, we also found that compared the baseline data with the non-SR group, the SR group had lower creatinine (64.8 ± 22.1 μmol/L vs. 74.0 ± 22.1 μmol/L, *P* = 0.006) and urea (5.2 ± 2.0 mmol/L vs.* 6.0* ± 2.3 mmol/L, *P* = 0.017); higher serum albumin level (31.0 ± 24.7 g/L vs. 25.1 ± 6.2 g/L, *P* = 0.038) and eGFR (103.8 ± 20.5 ml/min/1.73m^2^ vs. 97.0 ± 20.5 ml/min/1.73m^2^, *P* = 0.025) (Table 3).

Next, we compared genetic variants in terms of response to immunosuppressive therapy and renal outcomes. We found no statistically significant difference in response to immunosuppressive therapy. We also found that three risk genes for PMN development (A allele of rs2715918, A allele of rs4665143 and A allele of rs2187668) were not associated with renal outcomes and did not predict response to immunosuppressive therapy. However, we found that rs3828323 patients with one or two T alleles (CT/TT) had higher kidney survival than those without T allele (CC). Subgroup analysis also found that in the group of immunosuppression, the CT/TT genotype for rs3828323 achieved higher cumulative survival rate than CC genotype. It is suggested that the mutation of rs3828323 may be a potential target for immunosuppressive therapy of membranous nephropathy, and its mechanism deserves further study. In our previous study, rs3828323 was identified as a protective allele, indicating that a protective allele can not only reduce the susceptibility of PMN, but also serve as a predictor for real outcomes. Liu et al. found that two loci rs35771982 and 6,757,188 were associated with low remission rate after immunosuppressive therapy [15]. Bullich et al. revealed that patients with both rs2187668 AA/AG and rs4664308 AA had better therapeutic effect with immunosuppressive agents, and slower progression of renal disease [17]. Our results are not consistent with the previous research. There are several potential explanations. PMN is a complex disease with both genetic and environmental factors contributing to the progress of the disease. Genetic heterogeneity among different races, or environmental factors, can influence disease progression. Furthermore, various clinical conditions of PMN patients may also have an impact on the disease progression. Further studies with a large sample size and longer duration of follow up is needed to explore the impact of SNP on treatment.

Nonsynonymous SNP rs3828323 C > T within *PLA2R* (Gly1106Ser) is located in a coding region of exon 24 [16]. Previous literature and our study have confirmed that it is related to PMN susceptibility [16, 19, 23, 26, 27]. The SNP mutation sites of *PLA2R* can be found in both coding region or non-coding region, especially in intron or exon boundary region [16]. Any SNP may lead to different transcriptome lengths, which may lead to changes in protein function. A longer cut segment will form a stable transmembrane *PLA2R* protein, while a shorter cut segment will form a soluble enhanced *PLA2R* protein [28]. In conclusion, the SNP mutation of *PLA2R* gene may lead to the conformational change of *PLA2R*, and ultimately lead to the activation of *PLA2R* antibody, affecting the susceptibility and progress of the disease [23]. SNP mutation of *HLA-DQA1* is more dangerous to PMN than *PLA2R*. There is a combined effect between these two mutations. Some researchers believed that *HLA-DQA1* may be the dominant factor in the occurrence of PMN [13]. Its effect on the PMN autoantibody production may not only be through *PLA2R* pathway, but also through other ways [13]. In short, it seemed that the gene mutation of immunological immune system proteins (such as *HLA-DQA1*) is closely related to the gene mutation of glomerular component proteins (such as *PLA2R*), although the causal relationship between these two genes mutation is not yet clear. The direct relationship has not yet been established, but the whole mechanism is probably due to a complex system composed of trigger factors (immune system/*HLA-DQA1*), transduction pathway (*PLA2R* antibody) and target (glomerular antigen) related to [13]. Further researches on the occurrence and progress of SNP and PMN is needed.

Patients with the low-risk alleles of *PLA2R* and *HLA-DQA1* had the lowest positive rate for anti-*PLA2R* antibody (41.6%). In contrast, patients with the high-risk alleles of *PLA2R* and *HLA-DQA1* had the highest positive rate for anti-*PLA2R* antibody (87.8%). In all PMN patients who were tested for anti-*PLA2R* antibodies, treatment responses were seen in 71 patients. Among them, 57 patients had positive anti-*PLA2R* antibodies, of which 34 patients (59.6%) achieved spontaneous remission. Among 14 patients with negative anti-*PLA2R* antibodies, only 5 patients (35.7%) achieved spontaneous remission. Serum anti-*PLA2R* antibodies can be used in clinical diagnosis of PMN, especially in elderly patients with nephrotic syndrome or those who are not suitable for renal biopsy [29]. Anti-*PLA2R* antibody has high specificity and sensitivity. High titer of anti-*PLA2R* antibody indicates high risk of deterioration of renal function, low possibility of clinical remission, high recurrence rate and high risk of recurrence after renal transplant [9, 30, 31]. However, current literature reported the association between anti-*PLA2R* antibody and disease prognosis with inconsistent results [12, 23, 32]. suggesting that further higher quality research is needed in the future.

The major limitation of our study was the relatively short duration and wide range of follow up time and small sample size. We were unable to fully explore the association between anti-*PLA2R* autoantibodies and renal disease progression due to the retrospective nature of this study. The reason why we chose such a wide follow-up time range was that we would like to collect data using real-time approach in order to reflect the real clinical practice. In fact, some studies related to this topic also employed the same approach with wide follow-up time range. However, a wide time range may cause potential bias to the study. We hope to use these data to get preliminary results and provide a basis for more rigorous prospective research in the future.

## Conclusions

In conclusion, to our knowledge, this is the first study identified that patients with CT/TT genotype achieved higher cumulative survival rate than patients with CC genotype for rs3828323. Our study also showed that rs3828323 T allele was marginally significantly associated with hypertension. Urea was a risk factor, and eGFR and albumin were protective factors for non-remission after treatment for PMN.

## Supplementary Information

**Additional file 1**. The datasets used and analysed during the current study.

**Additional file 2**. Supplementary Tables for the current study.

## Data Availability

The datasets used and analysed during the current study are available under Additional file 1.

## Abbreviations

CR: Complete remission
ESRD: End stage renal disease
*HLA-DQA1*: Human leukocyte antigen complex class II HLA-DQα-chain 1
HWE: Hardy–Weinberg equilibrium
*PLA2R*: M-type phospholipase A2 receptor
PMN: Primary membranous nephropathy
PR: Partial remission
SNPs: Single nucleotide polymorphisms
SR: Spontaneous remission
