# Correction to: The association between variants in *PLA2R* and *HLA-DQA1* and renal outcomes in patients with primary membranous nephropathy in Western China

**DOI:** 10.1186/s12920-021-00992-1

**Published:** 2021-05-31

**Authors:** Shulei Fan, Qiuxia Wang, Amanda Y. Wang, Ping Zhang, Xiang Zhong, Shasha Chen, Guisen Li, Li Wang, Wei Wang

**Affiliations:** 1 Department of Nephrology and Institute of Nephrology, Sichuan Academy of Medical Science and Sichuan Provincial People’s Hospital, Chengdu, 610072 China; 2 The Renal and Metabolic Division, The George Institute for Global Health, University of NSW, Sydney, Australia; 3 The Department of Renal Medicine, Concord Repatriation General Hospital, Concord, NSW Australia; 4 The Concord Clinical School, University of Sydney, Camperdown, Australia; 5 Department of Respiratory Medicine, The Second Affiliated Hospital of Chongqing Medical University, Chongqing, China

## Correction to: BMC Med Genomics (2021) 14:123 https://doi.org/10.1186/s12920-021-00969-0

Following publication of the original article [1], the authors would like to add the following funding statement:

Amanda Y. Wang is funded by RACP Jacquot Research Establishment Award, Australia.

The original article [1] has been corrected.
