# Supplementary material for: The association between variants in PLA2R and HLA-DQA1 and renal outcomes in patients with primary membranous nephropathy in Western China
Source: BMC Med Genomics. 2021 May 8;14:123. doi: 10.1186/s12920-021-00969-0 (PMC8105990; doi:10.1186/s12920-021-00969-0)
Supplement: Supplementary file 2 — Additional file 2. Supplementary Tables for the current study. [file 12920_2021_969_MOESM2_ESM.docx]

**Supplementary tables**

**Supplementary Table S1 Association of alleles, genotype frequencies and different genetic models with 24h-u-pro in PMN patients**

| gene | Rs# | A/B | allele | genotype | dominant | recessive | additive |
| --- | --- | --- | --- | --- | --- | --- | --- |
|  |  |  | *P* | *P* | *P* | *P* | *P* |
| PLA2R1 | rs4664308 | A/G | 0.898 | 0.973 | 0.857 | 0.933 | 0.901 |
|  | rs3828323 | C/T | 0.803 | 0.607 | 0.577 | 0.549 | 0.812 |
|  | rs2715918 | G/A | 0.152 | 0.318 | 0.289 | 0.186 | 0.172 |
|  | rs6757188 | C/T | 0.765 | 0.849 | 0.985 | 0.601 | 0.787 |
|  | rs4665143 | G/A | 0.248 | 0.365 | 0.736 | 0.160 | 0.259 |
|  | rs35771982 | G/C | 0.926 | 0.880 | 0.810 | 0.721 | 0.928 |
|  | rs3749119 | C/T | 0.414 | 0.570 | 0.596 | 0.315 | 0.429 |
| HLA-DQA1 | rs2187668 | G/A | 0.501 | 0.476 | 0.345 | 0.570 | 0.477 |

**Supplementary Table S2 Association of alleles, genotype frequencies and different genetic models with eGFR in PMN patients**

| gene | Rs# | A/B | allele | genotype | dominant | recessive | additive |
| --- | --- | --- | --- | --- | --- | --- | --- |
|  |  |  | *P* | *P* | *P* | *P* | *P* |
| PLA2R1 | rs4664308 | A/G | 0.687 | 0.670 | 0.908 | 0.999 | 0.696 |
|  | rs3828323 | C/T | 0.133 | 0.353 | 0.209 | 0.999 | 0.162 |
|  | rs2715918 | G/A | 0.936 | 0.468 | 0.611 | 0.386 | 0.940 |
|  | rs6757188 | C/T | 0.618 | 0.902 | 0.681 | 0.730 | 0.652 |
|  | rs4665143 | G/A | 0.715 | 0.850 | 0.997 | 0.595 | 0.722 |
|  | rs35771982 | G/C | 0.453 | 0.669 | 0.589 | 0.999 | 0.465 |
|  | rs3749119 | C/T | 0.867 | 0.654 | 0.898 | 0.999 | 0.871 |
| HLA-DQA1 | rs2187668 | G/A | 0.507 | 0.431 | 0.340 | 0.999 | 0.483 |

**Supplementary Table S3 Association of alleles, genotype frequencies and different genetic models with blood pressure in PMN patients**

| gene | Rs# | A/B | allele | genotype | dominant | recessive | additive |
| --- | --- | --- | --- | --- | --- | --- | --- |
|  |  |  | *P* | *P* | *P* | *P* | *P* |
| PLA2R1 | rs4664308 | A/G | 0.686 | 0.519 | 0.975 | 0.293 | 0.694 |
|  | rs3828323 | C/T | 0.008^a^ | 0.029^b^ | 0.037^c^ | 0.034^d^ | 0.012^e^ |
|  | rs2715918 | G/A | 0.624 | 0.465 | 0.980 | 0.255 | 0.642 |
|  | rs6757188 | C/T | 0.501 | 0.826 | 0.557 | 0.673 | 0.542 |
|  | rs4665143 | G/A | 0.415 | 0.717 | 0.465 | 0.553 | 0.427 |
|  | rs35771982 | G/C | 0.968 | 0.489 | 0.685 | 0.352 | 0.969 |
|  | rs3749119 | C/T | 0.873 | 0.556 | 0.851 | 0.352 | 0.878 |
| HLA-DQA1 | rs2187668 | G/A | 0.199 | 0.395 | 0.181 | 0.629 | 0.176 |

corrected *P*-values (*P*c) were calculated using the Bonferroni inequality method

^a^*P*c=0.064, ^b^*P*c=0.232, ^c^*P*c= 0.296, ^d^*P*c=0.272, ^e^*P*c=0.096
